# Anticoccidial Vaccination Is Associated with Improved Intestinal Health in Organic Chickens

**DOI:** 10.3390/vetsci9070347

**Published:** 2022-07-09

**Authors:** Désirée S. Jansson, Johan Höglund, Elisabeth Bagge, Tomas Jinnerot, Magne Kaldhusdal

**Affiliations:** 1 Department of Animal Health and Antimicrobial Strategies, National Veterinary Institute (SVA), SE751 89 Uppsala, Sweden; elisabeth.bagge@sva.se; 2 Department of Clinical Sciences, Swedish University of Agricultural Sciences (SLU), SE750 07 Uppsala, Sweden; 3 Department of Biomedical Sciences and Veterinary Public Health, Section for Parasitology, Swedish University of Agricultural Sciences (SLU), SE750 07 Uppsala, Sweden; johan.hoglund@slu.se; 4 Department of Microbiology, National Veterinary Institute (SVA), SE751 89 Uppsala, Sweden; tomas.jinnerot@sva.se; 5 Department of Food Safety and Animal Health, Norwegian Veterinary Institute, N-1431 Ås, Norway; magne.kaldhusdal@vetinst.no

**Keywords:** anticoccidial vaccination, broiler, *Clostridium perfringens*, coccidiosis, *Eimeria* spp., necrotic enteritis, *netB*, organic production

## Abstract

**Simple Summary:**

In recent years, the number of organic chicken farms has increased. Chickens can be infected by single-cell parasites, coccidia, which cause lesions in the lining of the intestine leading to poor growth and sometimes death (coccidiosis). This infection can also lead to overgrowth in the intestine of a bacterium, *Clostridium perfringens*, that may cause further damage (necrotic enteritis). Prevention is often achieved by adding substances in the feed that will slow down the development of parasites and bacteria, but this is not allowed in organic farming. The aim of this study was to investigate if vaccination against coccidia can prevent these diseases in organic chickens. Vaccinated chickens developed milder gut lesions, had fewer and less damaging *C. perfringens*, and had similar or higher body weight compared to unvaccinated chickens six weeks after vaccination. No deaths from coccidiosis or necrotic enteritis occurred among vaccinated chickens while some unvaccinated chickens died from these diseases. We conclude that vaccination against coccidia benefits organic chickens. This study provides knowledge supporting further development of the organic chicken industry. The results are also of relevance to the management of coccidiosis and necrotic enteritis in conventional broilers.

**Abstract:**

*Eimeria* spp. and *Clostridium perfringens* (CP) are pathogens associated with coccidiosis and necrotic enteritis (NE) in broiler chickens. In this study we evaluated the effect of anticoccidial vaccination on intestinal health in clinically healthy organic Ross 308 chickens. On each of two farms, one unvaccinated flock (A1 and B1) was compared to one vaccinated flock (A2 and B2) until ten weeks of age (WOA). Faecal oocysts were counted weekly, and species were identified by PCR (ITS-1 gene). Lesion scoring, CP quantification and PCR targeting the CP NetB toxin gene were performed at three, four, and six WOA and chickens were weighed. Necropsies were performed on randomly selected chickens to identify coccidiosis/NE. Oocyst shedding peaked at three WOA in all flocks. Later oocyst shedding (*E*. *tenella*/*E*. *maxima*) in unvaccinated flocks at 5–7 WOA coincided with coccidiosis/NE. Although results differed somewhat between farms, vaccination was associated with lower intestinal lesion scores, reduced caecal CP counts, lower proportions of *netB*-positive CP, lower body weight at three–four WOA, and similar or slightly increased body weight at six WOA. In conclusion, the intestinal health of organic broilers can benefit from anticoccidial vaccination when oocyst exposure levels are high.

## 1. Introduction

Animal health and welfare are priority issues in organic broiler production. The combined effects of low stocking density, lower-protein diets, access to the outdoors, roughage supply, and use of slower-growing genotypes are expected to promote animal health and welfare. Organic broilers may, however, be at higher risk of infections than confinement-reared broilers since contact with pathogens transmitted from soil, water, and through wildlife contact is hard to avoid [1]. Free-range is also likely to be associated with increased risks for injury such as predation [2]. In addition, broiler genotype selection, feed composition, and management must be adapted to the prolonged rearing periods, organic husbandry methods, and variable conditions associated with weather and seasonal effects.

Enteric infections are associated with reduced welfare, increased mortality, low body weight (BW) gain, and impaired feed conversion in broiler production. The two most important enteric diseases are coccidiosis and necrotic enteritis (NE), which may manifest as sub-clinical or clinical disease. Coccidiosis is caused by protozoan parasites in genus *Eimeria*, which are ubiquitously present in most poultry farms. Necrotic enteritis is associated with selective proliferation of virulent strains of *Clostridium perfringens* (CP) [3]. This is a Gram-positive, spore-forming anaerobic bacterium and a normal component of the chicken gastrointestinal microbiota [4]. The pore-forming toxin NetB is considered a major virulence factor of CP in chickens [5,6]. In conventional broilers, NE may occur anytime from two weeks of age (WOA) until slaughter. Coccidiosis and NE are often, but not invariably, observed concomitantly, since *Eimeria*-induced lesions are associated with increased risks for development of NE [3]. Other examples of predisposing factors for NE include feed composition (grains containing high levels of indigestible non-starch polysaccharides and animal protein sources, e.g., fish meal), high stocking density, and immunosuppressive viral infections [3].

Anticoccidial drugs and antimicrobial growth promoters have been widely used in broilers to contain coccidia and CP but legal restrictions, organic production, and antimicrobial-free rearing concepts have put increasing pressure to reduce their use. Therefore, anticoccidial vaccination and supplementation with pre- and probiotics, phytogenics, and organic acids are increasingly investigated and applied. Earlier studies have suggested that coccidiosis and NE may be of considerable significance in organic broilers [7,8,9]. The occurrence of enteric diseases on organic farms is, however, rarely reported.

The organic broiler meat production represents <1% of the total broiler meat output in Sweden. Only recently, slower-growing genotypes became available, but at the time of this study fast-growing genotypes were reared on organic farms with an allowed growth rate of at maximum 50 g/day and slaughter at a minimum age of 70 days. Organic broilers are reared in Sweden according to regulations and principles set by the Swedish certification body KRAV, in line with regulations of the European Union (EC No 834/2007) and Organics International (IFOAM) standards. The maximum flock size is 4800 birds with a stocking density of ten birds/m^2^ indoors. From May to September, outdoor access to at least four m^2^ per chicken exceeding 12 h/day is formally required and in spring and autumn outdoor access is provided, weather and soil conditions permitting. The target marketing (slaughtered) weight at the time of the study was 1.5–2.2 kg. Organic broilers are predominantly fed diets based on organically grown feed sources with continuous roughage allowance. Use of synthetic amino acids, anticoccidial drugs, and growth-promoting antimicrobials is prohibited.

The study objective was to assess the impact of anticoccidial vaccination status (Paracox^®^-5 vet., MSD Animal Health) on the gastrointestinal health of organic broilers on two commercial farms, through analyses of *Eimeria* spp. oocyst shedding, caecal CP counts, proportion of *netB*-positive CP isolates, intestinal lesions, and occurrence of coccidiosis and NE.

## 2. Materials and Methods

### 2.1. Ethic and Clinical Trial Approvals

The Swedish Ethical Committee on Animal Research (protocols M376-12 and M7-14) approved procedures involving chickens. The Medical Products Agency (protocol 5.1-2014-9625) granted a clinical study on medicinal products approval.

### 2.2. Intestinal Health Concept

The concept ‘intestinal health’ in this study encompasses the following outcome variables: intestinal lesion scores (coccidiosis scores and NE scores), caecal CP counts and *netB* proportions in randomly sampled chickens, OPG counts and *Eimeria* species in randomly sampled faecal specimens, and intestinal lesions in dead or culled birds. Gizzard scores were not considered indicators of intestinal health but were included in the analyses because of their potential impact on production performance and their impact on intestinal CP counts. Body weight was included in the analyses as a complementary variable indicating the combined impact of intestinal health and gizzard health on this aspect of production performance.

### 2.3. Farms, Birds, Husbandry Practices, and Weighing

Two commercial organic broiler farms (A and B) in southern Sweden volunteered for this cohort study. Two flocks on each farm (A1 and A2 on farm A and B1 and B2 on farm B) were included from placement until slaughter at around 70 days of age (DOA). Flocks A1 and B1 were placed in early spring (March) followed by a second flock (A2 and B2) on each farm five weeks later. Each flock consisted of 4800 non-sexed Ross 308 chickens purchased from the same commercial non-organic hatchery. Flocks A1 and B1 remained unvaccinated while flocks A2 and B2 were vaccinated with Paracox^®^-5 vet., (Intervet), containing sporulated precocious oocysts of *E*. *acervulina* (strain HP, 500–650 oocysts/dose), *E*. *mitis* (strain HP, 1000–1300 oocysts/dose), *E*. *maxima* (strains CP and MFP, 200–260 and 100–130 oocysts/dose, respectively), and *E*. *tenella* (strain HP, 500–650 oocysts/dose). Five thousand doses were administered according to the manufacturer’s instructions by spray on feed at placement. Neither farm used other vaccines. An anticoccidial vaccine had been administered on farm A in some flocks ten years earlier. Animal health was assessed by farmers daily and injured/diseased birds were culled according to pre-existing farm routines. Ninety randomly selected chickens per flock and occasion were weighed at transfer between sheds and houses (see below) at 20–21 and 38–40 DOA (*n* = 360), and 20 randomly selected chickens per flock and occasion were weighed at lesion scoring (see below) at 3, 4, and 6 WOA (*n* = 240).

Chickens were grown according to KRAV organic regulations and pre-existing farm routines. On arrival, birds were placed in a starter shed on chopped straw, with mechanical ventilation and artificial light (16-h light:8-h dark schedule). The temperature was initially maintained at 31–33 °C, and then gradually decreased to 24 °C at three WOA. Between flocks, the starter sheds were cleaned with cold water (high pressure cleaning), left to dry for 7–10 days, followed by potassium peroxymonosulfate sodium chloride (Vircon^TM^ S, LANXESS, Cologne, Germany) disinfection (farm A). On farm B, the floor and walls were painted with limewater (Ca(OH)_2_). At three WOA, the birds were transferred to three (farm A) or two (farm B) mobile houses without mechanical ventilation or heating. In addition to natural light, an 8 h dark:16 h light artificial lighting programme was provided. Chopped straw was used as bedding material on the ground. The houses were located at a distance of 50–100 m from each other in an open field on pasture. Daytime access to pasture was provided from four WOA. On day 39 ± 1, the farmers transferred randomly selected chickens to one (farm A) or two (farm B) additional neighbouring mobile houses to provide more space. The first and second study flock on both farms were placed at a distance from each other to preclude contact. Hygiene barriers for change of footwear were in use in starter sheds and mobile houses.

Water and feed (organically certified broiler diets) were provided *ad libitum*. Feed intake was not recorded. The same recipes were used for the first and second flock on the respective farms. On farm A, a starter diet (24.3% crude protein, 12.3 MJ/kg) was fed until day 14, followed by grower diet 1 (18.2% crude protein, 11.3 MJ/kg) from day 15 to 28, and grower diet 2 (14.7% crude protein, 10.9 MJ/kg) from day 29 until slaughter. The main ingredients were wheat, oats, and maize, and fishmeal was included in the starter diet (10.7%). On farm B, the starter diet (19.5% crude protein, 12.0 MJ/kg) was supplemented with 100 kg fish meal during the first week. The main ingredients were wheat, oats, and peas. The starter ration was replaced by a grower concentrate (23.3% crude protein, 12.6 MJ/kg) on day 22 onwards mixed with silage, cereals, lupin, sunflower seeds, and maize fed on the litter bed.

### 2.4. Sampling and Analyses

**Figure 1:**
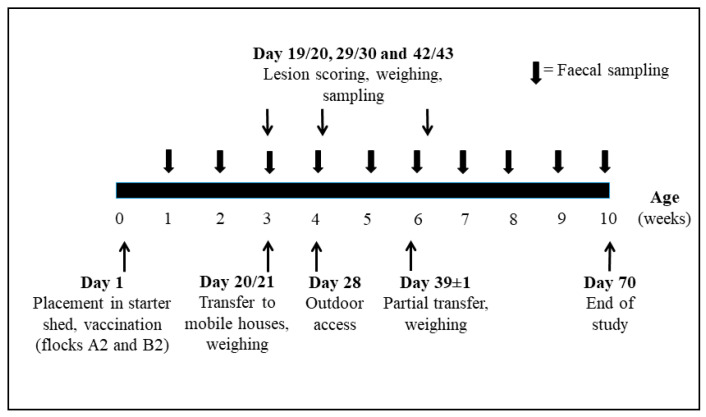
Experimental design and times of sampling.

Timing of sampling and weighing are shown in Figure 1.

#### 2.4.1. Faecal Sampling and Parasitological Analyses

Excreta (faecal) samples were collected weekly from one to ten WOA using plastic trays that were left on the litter bed overnight in starter sheds and in mobile houses except those that were only used from around 40 DOA. A total of 160 samples (four samples/flock and sampling occasion) were analysed by McMaster technique for detection of oocysts and helminth eggs (minimum detection level 50 oocysts/eggs per g faeces (opg/epg)). Samples were stored in 2% K_2_Cr_2_O_7_ solution at +4 °C pending further analysis. Sample preparation and conventional PCR for *Eimeria* spp. identification targeting the ITS-1 gene were performed as described [10] with minor modifications. Samples were sieved and washed in water by repeated centrifugation at 1800 rpm for 5 min. Oocysts were concentrated by saturated flotation and reconstituted with 100 µL water (Sigma-Aldrich, Chemie GmbH, Schnelldorf, Germany). Oocysts were mini-pestle ground, 5 µL proteinase K (20 mg/mL, Qiagen Inc, Hilden, Germany) was added, and the samples were vortexed and incubated at 56 °C for >3 h. Then, 50 µL of the supernatant was mixed with 50 µL GeneReleaser (BioVentures Inc., Murfreesboro, TN, USA), vortexed, and microwaved at full effect for six min, centrifuged at 13,500 rpm and supernatants were used as templates. Amplification was performed as described [10] with four (*E*. *acervulina*, *E*. *brunetti*, *E*. *maxima*) or eight (*E*. *mitis*, *E*. *necatrix*, *E*. *praecox*, *E*. *tenella*) pmol species-specific forward and reverse primers. Templates were used at 1:1 dilution, and negative samples were reanalysed using twice the amount of template, and at 1:2 and 1:10 dilutions. Positive and negative controls were used as described [10]. PCR products were analysed by electrophoresis, separated in 2% agarose gels, and stained with GelRed^TM^ nucleic acid stain (Biotium, Fremont, CA, USA) with a 50 bp ladder as size indicator (Fermentas, Vilnius, Lithuania).

#### 2.4.2. Lesion Scoring and Diagnostic Necropsy

At approximately three, four, and six WOA (days 19/20, 29/30, and 42/43), 20 clinically healthy chickens per flock (*N* = 240) were randomly selected for gross lesion scoring of gizzard and intestines. Chickens were euthanised by cervical dislocation and scored within a few minutes. Gross coccidial lesion scoring [11] was performed on a score 0 (no lesions) to 4 (severe lesions) scale for each intestinal segment. Gross intestinal lesions associated with NE were scored on a 0 (no lesions) to 6 (diffuse extensive necroses) scale [12,13]. Gizzard lesions were scored as described [14] with minor modification (scoring criteria 4 and 5 were combined and maximum attainable score was 22).

One dead or culled chicken per day and flock was randomly selected throughout grow-out for necropsy to assess whether and at what age coccidiosis and/or NE occurred. Coccidiosis and/or NE were only diagnosed if there were widespread and severe gross intestinal lesions (e.g., necroses, haemorrhage, caecal cores), microscopic confirmation (see below), and when no other cause of death or disease (in culled chickens) was observed. Most carcasses were frozen and thawed before examination. On days when no dead or culled birds were available, extra birds were collected the following day(s). For diagnostic confirmation, unstained wet smears of mucosal scrapings were used to detect and confirm presence of large numbers of coccidia (in fresh carcasses). Formalin-fixed tissues were investigated to confirm intralesional coccidia and lesions consistent with NE (in fresh and frozen carcasses). For histology, tissues were fixed in 10% phosphate-buffered formalin and were processed routinely followed by haematoxylin and eosin (HE) and Gram staining. Samples obtained at necropsy for detection of CP were cultured anaerobically on fastidious anaerobe agar plates (Lab M, Heywood, UK) at 37 °C and plates were assessed at 24 and 48 h of incubation.

#### 2.4.3. Enumeration of *C*. *perfringens*

After lesion scoring, one unopened caecum from each of the 20 birds examined per flock and sampling occasion was collected and transported on ice to the laboratory. From each bird, one sample of caecal contents (1–3 g) was homogenised in Stomacher bags and subjected to serial ten-fold dilution to 10^−7^ in 0.9% saline and was deep-spread in tryptose sulfite cycloserine agar (TSC, perfringens agar base, Oxoid, Basingstoke), UK) in duplicate, incubated anaerobically at 37 °C for 24 h, and colonies were counted. The lower detection limit was 10^2^ colony-forming units (CFU) per gram. Clostridial numbers were expressed as log_10_ CFU/g digesta. Five putative CP colonies/sample were subcultured on fastidious anaerobe agar plates (FAA, LabM), followed by culture on egg yolk agar (SVA). Species identification was achieved by colonial morphology, presence of a double zone haemolysis, and lecithinase positivity. Two randomly selected isolates/sample were stored in beef broth with 10% equine serum and 15% glycerol (SVA) at −70 °C.

#### 2.4.4. Analyses for the CP netB Gene and FAdV-1

DNA from single CP colonies prepared by boiling were used for real-time PCR detection of the *netB* as described [15]. PCR analysis to detect fowl adenovirus (FAdV) was performed on samples from both flocks because gizzard erosions were diagnosed at lesion scoring and at necropsy (see Section 3.1). The PCR detects an 897-base pair fragment containing the L1 loop of the hexon gene of FAdV and was performed as described [16] and visualised by 1% agarose gel electrophoresis. Amplicons were purified using the shrimp alkaline phosphatase-exonuclease I (ExoSapI) (U.S Biologicals, Swampscott, MA, USA) and nucleotide sequencing for FAdV species determination was performed using the BigDye Terminator v3.1 Cycle Sequencing Kit (Applied Biosystems, Foster City, CA, USA) according to the manufacturer’s instructions. The products were analysed on the ABI PRISM 3730xl genetic analyser (Applied Biosystems).

### 2.5. Feed Analyses

Feed samples from all starter and grower feed batches were analysed to detect ionophore coccidiostats (monensin, narasin, and salinomycin, detection limit 0.2 μm/kg feed) [17].

### 2.6. Statistical Analyses

Statistical analyses were performed using the Stata Software package (Release 17.0; College Station, StataCorp LP, College Station, TX, USA). The data were stratified with regard to chicken age and farm in order to control for the potential impact of these extraneous variables.

Summarisations of the outcomes and exposure (vaccinated or not) were used for the descriptive statistics. Body weights were stratified according to age, vaccination status, and farm. Statistical comparisons of vaccinated and unvaccinated birds were conducted using a two-sample mean-comparison *t*-test (*ttest* procedure) if the assumptions of normal distribution (Shapiro–Wilk normality test in the *swilk* procedure) and equal variances (Bartlett’s test in the *oneway* procedure) were fulfilled. If these assumptions were not fulfilled, a two-sample rank test (Kruskal–Wallis equality-of-populations rank test in the *kwallis* procedure) was used.

The effect of vaccination on OPG levels was examined using rank sum tests (Kruskal–Wallis equality-of-populations rank test-*kwallis* procedure, and the two-sample Wilcoxon rank sum test-*ranksum* procedure), controlling for an age effect and a potential farm effect (box plot, *graph box* procedure).

Coccidiosis lesion scores for all four intestinal segments were summed up into a total coccidiosis score for each bird. The effect of vaccination on intestinal scores and gizzard scores was examined (Kruskal–Wallis equality-of-populations rank test, *kwallis* procedure), controlling for an age effect and a potential farm effect (box plot, *graph box* procedure).

The effect of vaccination on CP counts was examined using a rank sum test (Kruskal–Wallis equality-of-populations rank test-*kwallis* procedure) controlling for potential effects of age and farm on CP counts (box plot, *graph box* procedure). In order to control for potential effects of farm and age on CP counts, data from day 20 on farm B were omitted when the effect of vaccination was examined. The effect of vaccination on *netB* proportions was examined using multivariable logistic regression analysis (*logistic* procedure).

## 3. Results

### 3.1. Animal Health, Post-Mortem Findings, and Impact of Vaccination on Body Weight

According to farmers, all four flocks appeared clinically unremarkable except for increased mortality during the first week of life in flocks A1 and B1 when *E*. *coli* infection was diagnosed (routine diagnostics, not included in study), and in flock B1 there was an incident of red fox predation on pasture. No birds/flocks received antimicrobial treatment.

A total of 274 chickens (number/flock: A1 = 68; A2 = 67; B1 = 66, B2 = 73) were examined *post-mortem* to determine if and at what age coccidiosis and/or NE were the cause of death or culling. Of these, 49% (range 43–56%) were found dead and the remaining chickens had been culled. Coccidiosis was diagnosed based on presence of necroses and/or haemorrhage and microscopic evidence of large numbers of coccidia. Caecal coccidiosis was diagnosed in three chickens in the unvaccinated flock A1 in 33–35-day-old birds, and in flock B1 caecal coccidiosis and/or small intestinal coccidiosis was diagnosed in nine chickens between 43 and 54 DOA. Microscopic findings consistent with NE, i.e., severe jejunal/ileal necroses, mononuclear inflammation, and aggregates of large Gram-positive rod-shaped bacteria adjacent to lesions, were observed in two chickens concurrent with small intestinal coccidiosis in the unvaccinated flock (at 49 and 50 DOA, respectively) on farm B (B1). Coccidiosis was not diagnosed among vaccinated birds in flocks A2 and B2.

Gross gizzard lesions were observed in 41 out of 66 chickens (62%) in flock B1 and in 35 out of 73 chickens (48%) in flock B2. Gizzard erosion was a rare finding in chickens from farm A. The earliest gizzard lesions were observed in one-week-old chickens on both farms and were observed throughout grow-out on farm B. Lesions ranged from focal discoloration and superficial pits and fissures to separation of the degraded koilin layer from the epithelium, inflammatory exudate, and extensive erosions and ulcers. Affected birds were often thin and undigested feed was present in ileum and colon in birds with extensive lesions. Microscopically, the koilin layer appeared less homogenous than normal with remnants of koilin rodlets separated by spaces filled with trapped degenerated inflammatory cells, groups of desquamated degenerated glandular epithelial cells, and fibrin. In 12 out of 30 birds examined by microscopy, large Gram-positive rod-shaped bacteria were observed in scattered aggregates or diffusely on the surface and within the koilin layer at varying numbers. There was a diffuse mononuclear infiltrate admixed with heterophils in lamina propria, at times of transmural extent. Infection with FAdV was considered a possible cause, despite absence of epithelial inclusion bodies.

Data on BW and the impact of vaccination on BW are shown in Table 1. Control for the farm effect on BW was possible at approximately three WOA. Statistical analyses indicated no significant effect of vaccination on BW on farm A on days 19 and 20. A statistically significant (*p* = 0.00, Kruskal–Wallis test) and 12% negative impact of vaccination on BW was detected on farm B at three WOA. At four WOA the impact of vaccination could only be assessed on farm A. At this age, vaccination appeared to reduce mean BW with 10% (*p* = 0.04, Kruskal–Wallis test). Due to the structure of the data, the impact of vaccination on BW could not be assessed separately per farm after four WOA. An analysis based on birds from both farms on days 42–43 indicated similar BW in both vaccination groups (*p* = 0.35, Kruskal–Wallis test), although with an estimated 7% higher mean BW in the vaccinated group.

### 3.2. Impact of Vaccination on Oocyst Shedding and Eimeria spp. Detection

**Figure 2:**
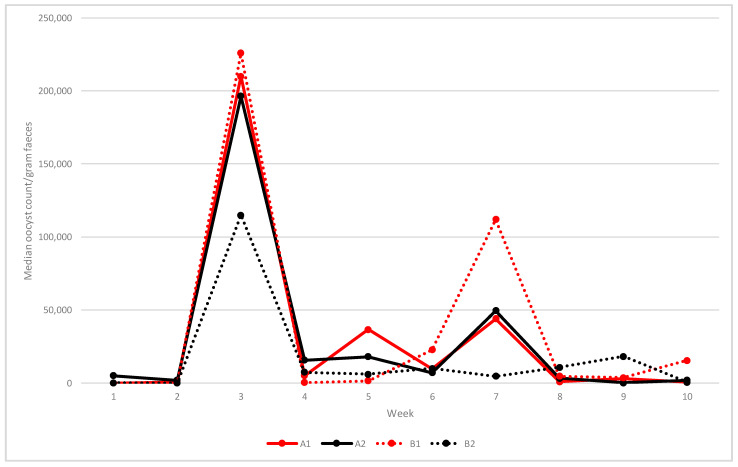
Number of oocysts per gram faeces (median of four samples) of four flocks of organic commercial broiler chickens on two farms (A and B) sampled weekly between one and ten weeks of age. Flocks A1 and B1 were not vaccinated. Flocks A2 and B2 were vaccinated at placement with an attenuated anticoccidial vaccine.

Median oocyst shedding at weekly intervals is shown in Figure 2. In unvaccinated flocks (A1 and B1), the first oocysts were detected at two WOA and shedding continued throughout grow-out. A first peak occurred in the starter sheds at three WOA with median opg levels of 210,000 (A1) and 226,000 (B1). Median oocyst counts then varied between around 36,750 and 44,000 opg between weeks 5 and 7 in flock A1, whereas a more distinct peak around 112,000 opg was observed at 7 weeks in flock B1.

In vaccinated flocks A2 and B2, low numbers of oocysts were present at one WOA (median opg 5200 in A2 and 200 in B2). Flock A2 showed a similar shedding pattern and opg levels as in unvaccinated flock A1 (median opg at three WOA 196,500 and 49,500 at seven WOA), but lower shedding at five WOA (18,000 opg) (Figure 2). In the vaccinated flock B2, the median oocyst shedding at three WOA was 115,000 opg, after which shedding remained at low levels (Figure 2).

Vaccination was associated with increased OPG counts during the first two weeks post-hatch, mostly because of higher counts in the vaccinated group (combined results from both vaccinated flocks) at one week of age (*p* < 0.01, Kruskal–Wallis test). No statistically significant difference in OPG counts between the two vaccinated flocks was demonstrated between 3–10 WOA.

PCR identification of *Eimeria* spp. is shown in Table 2. In flock A1, *E*. *tenella*, *E*. *maxima* and *E*. *acervulina* alone or in combinations were identified at 5–9 WOA. In flock B1, 2–5 *Eimeria* spp. were identified between six and ten WOA including *E*. *tenella*, *E*. *maxima*, *E*. *praecox,* and *E*. *acervulina*, with the addition of *E*. *necatrix* at ten WOA. In vaccinated flock A2, up to five *Eimeria* spp. were detected by PCR (Table 2). From seven WOA, oocysts could not be identified. In the vaccinated flock B2 different combinations of five *Eimeria* spp. were detected (Table 2). Nematode eggs were not detected in any study flock.

### 3.3. Impact of Vaccination on Lesion Scores

**Figure 3:**
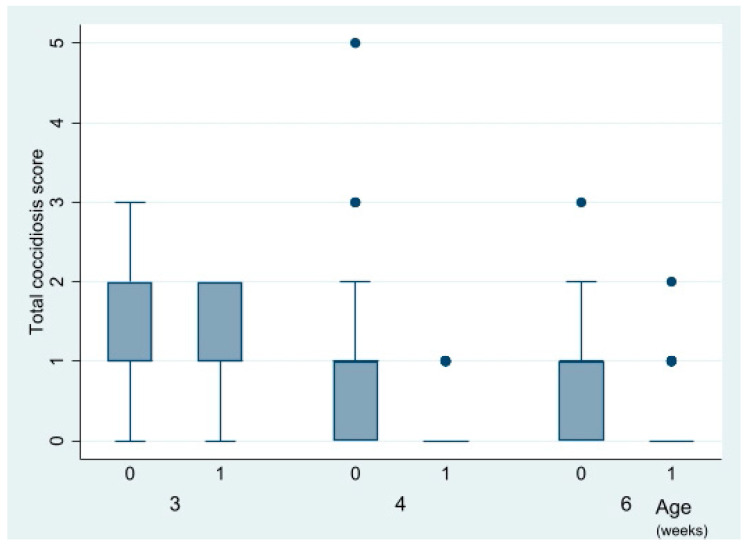
Box plot distribution of total intestinal coccidiosis scores (*Y*-axis) plotted against vaccination status (0 = not vaccinated flocks A1 and B1; 1 = vaccinated flocks A2 and B2) and age at lesion scoring in organic chickens. Dots represent outliers.

Coccidiosis lesions were detected in 54% of the examined chickens, and in all intestinal segments except colon (Table 3). Lesions were most prevalent in the duodenum (29% of birds) and the jejunum (27% of birds). Duodenal scores were generally low at four and six weeks of age. The total coccidiosis scores and the jejunal scores, but not the duodenal scores, were reduced (*p* = 0.00) by vaccination at four and six weeks of age, but not at three weeks of age (Figure 3). The median total coccidiosis score was reduced from 1 among unvaccinated to 0 among vaccinated chickens. This impact of vaccination was largely independent of farm. Intestinal NE lesions were not detected in any of the examined chickens at lesion scoring (for results of dead/euthanised chickens see Section 3.1).

**Figure 4:**
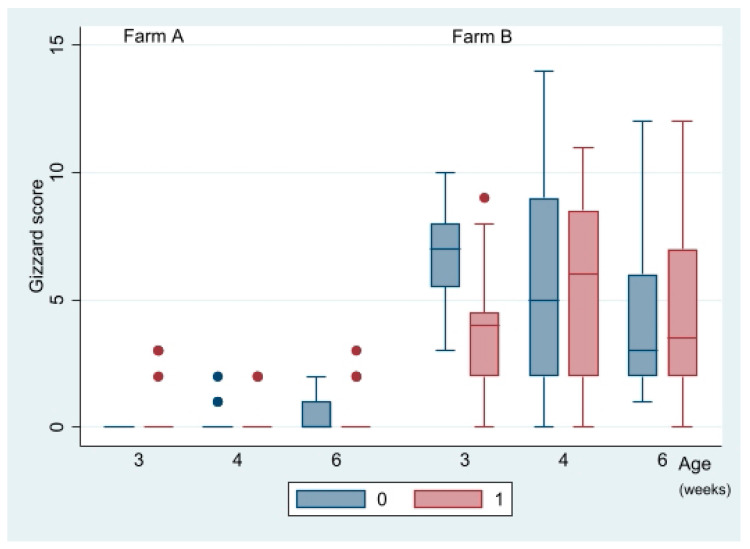
Box plot distribution of gizzard scores in organic chickens plotted against vaccination status (blue bars = not vaccinated, red bars = vaccinated), age at lesion scoring, and farm (farm A, farm B). Dots represent outliers.

Mean gizzard scores in relation to vaccination and farm are shown in Table 4. Gizzard scores above 3 were detected only on farm B, where 25% of the examined chickens were given a score of 12 or more. Thus, gizzard lesions were a problem on farm B and the effect was independent of age. There was no overall impact of vaccination on gizzard scores (*p* = 0.31, Kruskal–Wallis test). Examination of the interplay between vaccination, farm, and age suggested that vaccination was associated with reduced gizzard scores on farm B at three weeks of age (Figure 4) (*p* = 0.00, Kruskal–Wallis test). There was no significant impact of vaccination on gizzard scores on farm A or on farm B at weeks 4 and 6.

### 3.4. Impact of Vaccination on Clostridium perfringens Counts and netB PCR Proportions

**Figure 5:**
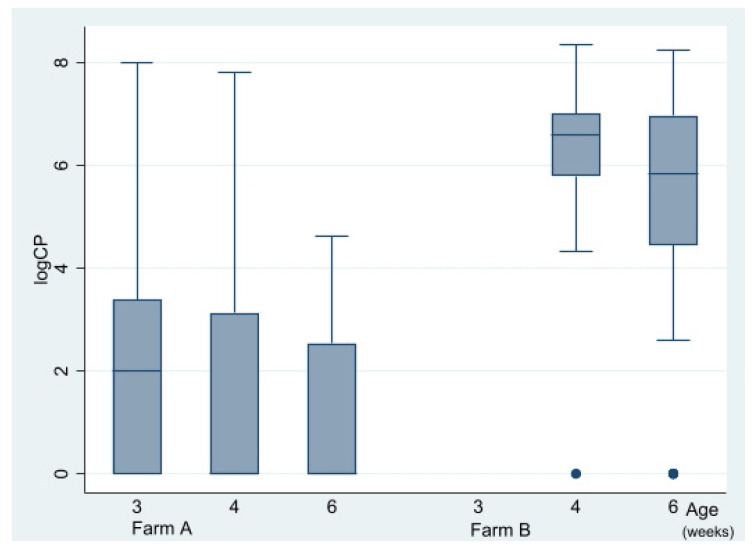
Box plot describing the distribution of *Clostridium perfringens* counts (logCP) per age at lesion scoring and farm (farm A, farm B) with organic broiler chickens. Unvaccinated and vaccinated birds were combined. Three-week-old chickens from farm B were excluded because of lack of data from unvaccinated birds. Dots represent outliers.

**Figure 6:**
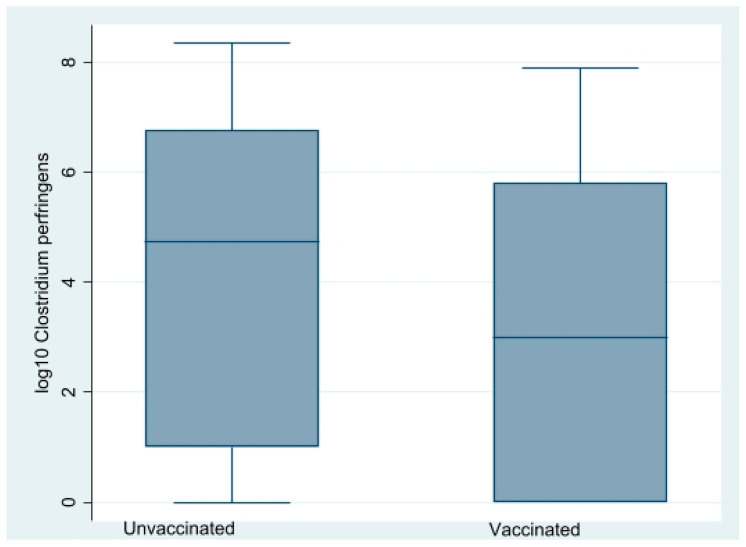
Box plot describing the effect of vaccination with an attenuated anticoccial vaccine on caecal log_10_ *Clostridium perfringens* counts (logCP) in four- and six-week-old organic broiler chickens. Combined data from farm A and B.

Caecal CP counts at the time of lesion scoring at three, four, and six WOA are shown in Figure 5. In total, 148 out of 220 birds were culture-positive (≥100 CFU/g). CP counts were obtained from all sampling occasions except from flock B1 at 20 DOA (due to a technical error) and varied substantially with age and farm (Figure 5). There was an association between farm and CP counts. Median CP counts of farm A and B were <100 and 1850,000, respectively (*p* = 0.00) during four to six WOA, Kruskal–Wallis test). A reducing impact (*p* = 0.01, Kruskal–Wallis test) of vaccination on CP counts on both farms was detected during four to six WOA (Figure 6). Separate analyses per farm indicated that this was independent of farm. On farm A, vaccination-reduced CP counts in all three age groups combined significantly (*p* = 0.00, Kruskal–Wallis test). At three WOA the impact of vaccination on CP counts could only be examined on Farm A, where vaccination also was associated with reduced CP counts (*p* = 0.01, Kruskal–Wallis test).

A total of 257 CP isolates were recovered in pure culture from 135 out of 148 culture-positive birds. Among the 123 birds with two isolates, five (4%) showed different results with *netB* PCR.

A difference in *netB*-proportions was found when comparing farm A (56% *netB* at four and six WOA birds) and farm B (5% *netB* at four and six WOA) (Table 5). The impact of farm at three WOA could not be evaluated due to a lack of data from unvaccinated chickens from farm B. Comparison of vaccinated and unvaccinated chickens at four or six WOA from both farms suggested a reducing impact of vaccination (9 and 23% *netB*-positive CP isolates from vaccinated and unvaccinated chickens, respectively (Table 5). The odds of presence of *netB* among isolates from these chickens, controlling for farm and age effects, were lower for vaccinated birds compared to unvaccinated birds (OR = 0.25, 95% CI = 0.08–0.82, *p* = 0.02). The odds of presence of *netB* on farm A during the whole study period, controlling for an age effect, were lower for vaccinated birds compared to unvaccinated birds (OR = 0.04, 95% CI = 0.01–0.17, *p* = 0.00).

### 3.5. Gizzard Cultures and Molecular Tests

Gizzard samples from two 20-day-old chickens from flock B1 were cultured anaerobically. Both revealed growth of CP of which one was *netB*-positive by PCR. The partial FAdV hexon gene was detected by PCR in all seven birds with gizzard erosion (samples from gizzard and caecal tonsils) representing all four flocks sampled at three or four WOA. Sequencing detected FAdV species D and E.

### 3.6. Feed Analyses

Ionophores were not detected in any of the feed samples.

## 4. Discussion

In this paper we describe the effects of a single round of anticoccidial vaccination on gastrointestinal health in clinically healthy organic broiler chickens on two commercial farms. Live anticoccidial vaccines are increasingly being used in broilers to prevent coccidiosis on organic farms and on conventional farms in some countries such as Norway [18] and in the USA [19], but the effects of vaccination of organic broilers on commercial farms has yet received little research attention.

In this study, anticoccidial vaccination was associated with increased oocyst shedding during the first two weeks post-hatch, probably originating from vaccine strains. After this, vaccination had no significant effect on oocyst shedding during the following eight weeks of the rearing period. This included the early oocyst peak shedding (Figure 2) at three WOA when *E*. *acervulina* was detected in three out of four flocks. Nevertheless, vaccination reduced coccidial lesion scores at four and six WOA, but not at three weeks. Together, and in combination with no diagnosed cases of coccidiosis among dead and culled chicken from vaccinated flocks, these results suggest that vaccination induced partially protective immune responses against *Eimeria*-induced lesions from around four WOA. These findings support previous reports from field studies in non-organic broilers [20,21].

Typically, *E. acervulina, E. maxima* and *E. tenella* predominate in young broiler chickens [10,22]. This was also the case in our study, regardless of vaccination status (Table 2). At lesion scoring, the gross appearance and location of lesions in duodenum, jejunum, and caeca also supported this finding. Moreover, in the unvaccinated chickens that died from coccidiosis, in some cases in combination with NE, severe necroses and hemorrhage were observed in jejunum and caeca, presumably associated with *E*. *maxima* and/or *E*. *tenella*.

Interestingly, other coccidia not included in the vaccine such as *E*. *brunetti* (flock A2), *E*. *necatrix* (flocks A2, B1, and B2), and *E*. *praecox* (flocks A2, B1, and B2) were detected by PCR during the later stages of grow-out (Table 2). Unfortunately, as lesion scoring was only done up until six WOA it is unclear whether they induced lesions. It cannot be ruled out that they contributed to mortality from coccidiosis. *Eimeria necatrix* [22] is a highly pathogenic species which is typically associated with coccidiosis in birds that are grown to a higher age than conventional broilers [22]. Similarly, *E*. *brunetti* is not typically a cause of coccidiosis in young broiler chickens and *E*. *praecox* is not considered to be highly pathogenic. Their occurrence was likely associated with the longer grow-out period in organic chickens compared to conventionally reared broilers. Another interesting finding in this study was that *E*. *tenella* was not detected after three WOA in vaccinated chicken while it was present in unvaccinated flocks between 5 and 8 WOA (Table 2). This suggests that vaccination protected birds from mortality from caecal coccidiosis.

Another aim of this study was to investigate how anticoccidial vaccination affected caecal CP counts. Vaccination was associated with reduced caecal CP counts in all three age groups when sampling was performed at lesion scoring at three, four, and six WOA on farm A and at four and six WOA (lack of complete data from three WOA) on farm B. Significant reductions in intestinal CP counts following anticoccidial vaccination of broilers have been shown previously in chickens co-challenged with CP and coccidia [23,24]. CP counts and gizzard scores were clearly higher on farm B than on farm A, independent of age and vaccination status. The high CP counts on farm B were most likely associated with the higher occurrence and severity of gizzard erosions on this farm [25].

A reducing impact of vaccination on gizzard lesions does not appear likely, because *Eimeria* spp. are not considered a risk factor for gizzard erosions. Furthermore, vaccination reduced CP counts on farm A even though gizzard erosions were few and very mild on this farm. CP counts at or above 100,000 per gram caecal content were found in 13% of the unvaccinated chickens on farm A, as opposed to in none (<2%) of the vaccinated (data not shown). In contrast, intestinal coccidia is a well-established predisposing factor for NE [3], and thus a condition associated with increased CP counts [3]. This predisposing effect is presumed to be due to intestinal changes induced by the coccidia that favour proliferation and toxin production by CP [3]. Chickens with NE may harbour high intestinal CP numbers (10^7^–10^8^ CFU/g) while healthy birds have significantly lower numbers (up to around 10^5^ CFU/g intestinal content) [26]. The data from farm A thus provide an example of an *Eimeria*-influenced CP level and suggests that the number of CP was too low to induce a detectable level of NE. However, it was high enough to be reduced by anticoccidial vaccination. A previous study suggested that higher caecal numbers of CP is present in organic broilers compared to chickens raised on conventional farms [4], possibly related to ionophore use in the latter. Moreover, dietary fishmeal and *Eimeria* infection induce major shifts to the intestinal microbiota, which could speculatively increase the risk of NE in organic chickens [27]. Despite that several of these predisposing factors were present in the study flocks and that CP counts were high on farm B, no signs of NE were observed except in two chickens concurrently infected with coccidia in flock B1.

Vaccination reduced *netB*-proportions on farm A but not on farm B. A possible explanation of this difference in vaccination effect on the two farms might be that the proportion of *netB* was clearly higher on farm A (56%) than on farm B (4%) (data from four and six WOA available). A potential effect on *netB* proportion is less likely to be detected when the proportion is low. We propose that the difference in *netB* proportion on the two farms was due to different origins of CP isolates examined from farm A and B. We also suggest that isolates from farm A originated to a larger extent from CP proliferation associated with intestinal coccidiosis, and that isolates from farm B originated to a larger extent from proliferation associated with gizzard erosions. Furthermore, based on this presumption, we hypothesise that CP that proliferated in the intestine had higher *netB* proportions than CP that proliferated in the gizzard. Thus, CP from gizzard erosions and CP from intestinal lesions may represent two distinct populations regarding *netB*, but further studies are needed.

Vaccination was associated with reduced BW at three (farm B) or four (farm A) WOA. At six WOA vaccination tended to increase BW, but the difference was statistically non-significant (Table 1). Caution is warranted in the interpretation of these results, because although measures were taken to minimise the risk of confounding effect of age, this risk could not be completely eliminated. These findings suggest that the impact on BW of anticoccidial vaccination depended on the time elapsed after vaccination. Reduced BW at three to four WOA might partly be related to vaccine strains, which might to some extent impair intestinal function during the first weeks after hatch. Another factor of possible significance is the energy-demanding earlier immune response induced by the vaccine. A third factor could be the high proportion of chickens that were exposed to vaccine strains as from day of hatch, as opposed to the more random colonisation by *Eimeria* spp. from the farm environment. BW data from six WOA indicate similar or higher BW in vaccinated broilers compared to unvaccinated chickens, which suggests that weight gain lost in the vaccine group up to four WOA had at least been compensated for by six WOA. A likely explanation for this development is exposure to non-attenuated strains of *Eimeria* spp.

By performing lesion scoring and *post-mortem* examinations we aimed to determine whether coccidiosis and NE caused mortality and/or culling in unvaccinated and vaccinated chickens and, if so, to identify age intervals of increased susceptibility. Due to the way chickens were selected, conclusions on incidence cannot be inferred. No signs of NE were observed at lesion scoring regardless of farm and vaccination status. Nevertheless, coccidiosis and NE were diagnosed concurrently in two unvaccinated chickens from flock B1, which supports previous reports that both diseases may cause mortality in commercial unvaccinated organic broilers [7,8,9]. There is a general lack of published information on intestinal health in commercial organic broiler flocks. In a recent paper from Sweden, up to 44% of organic chickens on commercial farms showed signs of diarrhoea between 44 and 62 DOA [28]. Reasons for this finding were not investigated, but it suggests that enteric disturbances occur on organic farms, which justifies further investigation. When clinical coccidiosis occurs in commercial organic broiler flocks it is likely a result of gradual build-up of residual oocysts in litter and/or on pasture. Necrotic enteritis can follow but can also develop independently. An anticoccidial vaccination program would most likely prevent most such mortality events.

The high occurrence of gizzard erosion on farm B (Figure 4, Table 5) was an unexpected finding in this study which may have interfered with and obscured the effects of vaccination outcome. Moreover, it was a likely cause of the low BWs of chickens on farm B. A multitude of aetiologies have been suggested for gizzard erosion including starvation, combined effects of pelleted feed and lack of litter, nutritional deficiencies, mycotoxins, dietary biogenic amines, and infection with fowl adenovirus (FAdV) serotype 1/species A [29]. A viral aetiology seemed less plausible in this study as FAdV species A was not detected but cannot be dismissed. Other dietary factors might also have caused the high occurrence of gizzard erosions on farm B, but the effect of feed and feeding on gizzard integrity is poorly understood and warrants closer study.

## 5. Conclusions

Anticoccidial vaccination was associated with lower coccidial lesion scores, reduced caecal CP counts, lower proportions of *netB*-positive CP isolates, lower body weight at three to four WOA, and similar or slightly increased BWs at six WOA. These results suggest that organic chickens may benefit from anticoccidial vaccination. The impact of vaccination on BW was age dependent.

## Figures and Tables

**Table 1:** Summary of body weight (BW) data at different ages (19–43 days of age), vaccination status (0 = unvaccinated or 1 = anticoccidial vaccination), and farm of origin (farm A or B) from randomly selected organic chickens. Bird groups of 20 were weighed at lesion scoring, and groups with 85–90 birds were weighed at transfer of chickens during the rearing period.

		Farm A				Farm B			
Age (Days)	Vacci-Nated	Birds (*n*)	Mean/Median BW	% BW ^3^	*p*-Value	Birds (*n*)	Mean/Median BW	% BW ^3^	*p*-Value
19	0	20	593/578		*p* = 0.20 ^1^	0	-		
	1	20	558/557	94/96		0	-		
20	0	90	632/632		*p* = 0.87 ^2^	20	351/389		*p* = 0.08 ^2^
	1	90	639/633	101/100		20	302/306	86/79	
21	0	0	-			90	381/386		*p* = 0.00 ^1^
	1	0	-			90	336/336	88/87	
29	0	20	1007/1001		*p* = 0.04 ^1^	0	-		-
	1	20	910/889	90/89		20	516/505		
30	0	0	-			20	539/556		-
	1	0	-			0	-		
38	0	90	1463/1440		-	0	-		-
	1	0	-			0	-		
39	0	0	-		-	90	1032/995		-
	1	90	1480/1485			0	-		
40	0	0	-		-	0	-		-
	1	0	-			85	1169/1175		
42	0	20	1438/1439		-	0	-		-
	1	0	-			20	1076/1051		
43	0	0	-		-	20	959/970		-
	1	20	1522/1503			0	-		
			Farm A + B						
Age	Vacci-nated	Birds	Mean/median BW		*p*-value			% BW ^3^	Mean age
19–	0	220	500/492		*p* = 0.09 ^2^				20.35
21	1	220	477/452					95/92	20.35
42–	0	40	1199/1232		*p* = 0.35 ^1^				42.50
43	1	40	1279/1244					107/101	42.50

^1^ Two-sample mean-comparison *t*-test, ^2^ Kruskal–Wallis equality-of-populations rank test, ^3^ Relative mean/median body weight of vaccinated birds, expressed as percentage of unvaccinated in the same age group.

**Table 2:** PCR identification of *Eimeria* spp. oocysts in four flocks of commercial broiler chickens on two organic farms (A and B) sampled weekly between one and ten weeks of age. Flocks A2 and B2 were vaccinated at placement with an attenuated anticoccidial vaccine. Abbreviations: NA = not available; *ace* = *E*. *acervulina*, *max* = *E*. *maxima*, *ten* = *E*. *tenella*, *bru* = *E*. *brunetti*, *nec* = *E*. *necatrix*, *pra* = *E*. *praecox*, *mit* = *E*. *mitis*.

Age (Weeks)	Unvaccinated Flocks	*ace*	*max*	*ten*	*bru*	*nec*	*pra*	*mit*	Vaccinated Flocks	*ace*	*max*	*ten*	*bru*	*nec*	*pra*	*mit*
1	A1	NA	NA	NA	NA	NA	NA	NA	A2	-	-	-	-	-	-	-
2		-	-	-	-	-	-	-		+	-	-	-	-	-	-
3		-	-	-	-	-	-	-		+	-	-	-	-	-	-
4		-	-	-	-	-	-	-		+	-	-	-	-	-	-
5		-	-	+	-	-	-	-		+	+	-	-	-	-	-
6		-	+	+	-	-	-	-		+	+	-	-	-	+	-
7		+	+	+	-	-	-	-		+	+	-	+	+	+	-
8		-	-	-	-	-	-	-		-	-	-	-	-	-	-
9		+	-	-	-	-	-	-		-	-	-	-	-	-	-
10		-	-	-	-	-	-	-		-	-	-	-	-	-	-
1	B1	NA	NA	NA	NA	NA	NA	NA	B2	-	-	-	-	-	-	-
2		-	-	-	-	-	-	-		+	-	-	-	-	-	-
3		+	-	-	-	-	-	-		+	-	+	-	-	-	-
4		+	-	-	-	-	-	-		-	-	-	-	-	-	-
5		+	-	-	-	-	-	-		+	+	-	-	-	-	-
6		-	+	+	-	-	+	-		-	+	-	-	-	+	-
7		+	+	+	-	-	+	-		+	+	-	-	+	+	-
8		-	+	+	-	-	-	-		+	+	-	-	+	+	-
9		+	+	-	-	-	-	-		+	-	-	-	-	-	-
10		+	+	-	-	+	+	-		+	-	-	-	-	-	-

**Table 3:** Mean coccidial lesion scores in relation to vaccination status and farm identity at three, four, and six weeks of age in four organic commercial broiler flocks. Twenty randomly selected clinically healthy chickens were scored per flock on each sampling occasion. Total mean: combined mean score of all combined observations from all three intestinal segments collected on all three sampling occasions. All age groups: mean score of observations combined for all age groups but presented separately per intestinal segment. Scores per week of age: mean score of observations combined for all intestinal segments but presented separately per week of age. Abbreviations: duo = duodenum, jej = jejunum, cae = caecum.

	Age at Sampling (Weeks)															
Vaccination/Farm	3			4			6			Total Mean	All Age Groups			Scores per Weeks of Age		
Segment/age (Weeks)	duo	jej	cae	duo	jej	cae	duo	jej	cae		duo	jej	cae	3	4	6
Vaccinated	1.3	0.1	0	0	0.2	0	0	0.2	0	0.2	0.4	0.2	0	0.5	0.1	0.1
NOT vaccinated	1.3	0.1	0	0.3	0.6	0.	0	0.9	0.1	0.4	0.5	0.5	0.1	0.5	0.3	0.3
Farm A	1.0	0.1	0	0.2	0.4	0.1	0	0.2	0	0.22	0.4	0.2	0	0.4	0.2	0.1
Farm B	1.0	0.1	0	0.1	0.3	0	0	0.6	0.1	0.23	0.4	0.3	0	0.4	0.1	0.2

**Table 4:** Mean gizzard lesion in relation to vaccination status and farm identity at three, four, and six weeks of age in four organic commercial broiler flocks. Twenty randomly selected clinically healthy chickens were scored per flock on each sampling occasion.

	Age at Sampling (Weeks)			
Vaccination/Farm	3	4	6	All Age Groups
Vaccinated	2.1	2.7	2.4	2.4
NOT vaccinated	3.4	2.9	2.4	2.9
Farm A	0.2	0.2	0.5	0.3
Farm B	5.3	5.4	4.3	5.0

**Table 5:** Results of *netB* PCR analysis of *Clostridium perfringens* isolates in relation to vaccination status and farm identity at three, four, and six weeks of age (WOA) in four organic commercial broiler flocks at bird and isolate levels. Twenty randomly selected clinically healthy chickens from four flocks were scored per flock and sampling occasion.

	Isolates (*n*)		Total Isolates (*n*)	NetB + Isolates (%)
	NetB+	NetB-		
Vaccinated both farms 4–6 WOA	7	74	81	9
Unvaccinated both farms 4–6 WOA	23	75	98	23
Sum 4–6 WOA	30	149	179	17
Vaccinated farm A 3–6 WOA	4	23	27	15
Unvaccinated farm A 3–6 WOA	39	13	52	75
Farm A 4–6 WOA	24	19	43	56
Farm B 4–6 WOA	6	130	136	5
Sum	30	149	179	17

## Data Availability

The data presented in this study are available on reasonable request from the corresponding author. The data are not publicly available due to privacy restrictions.
